# A Cloudy Conical Cornea

**DOI:** 10.5811/cpcem.2017.9.35314

**Published:** 2018-01-09

**Authors:** Katherine Biggs, Sean Stuart

**Affiliations:** Naval Medical Center Portsmouth, Department of Emergency Medicine, Portsmouth, Virginia

## CASE PRESENTATION

A 26-year-old Black male presented with right eye redness, discomfort and decreased vision over the preceding two weeks. There was no history of trauma or other precipitating event. Physical exam included acuity of 20/200 in the right eye, bilateral conjunctival injection, normal pupillary appearance and reactivity, and full, pain-free movement of bilateral orbits. An opacification was noted along the margin of the right iris extending into the visual field, without visualized defect of the outer cornea or fluorescence uptake on slit lamp examination.

## DISCUSSION

**Image 1:**
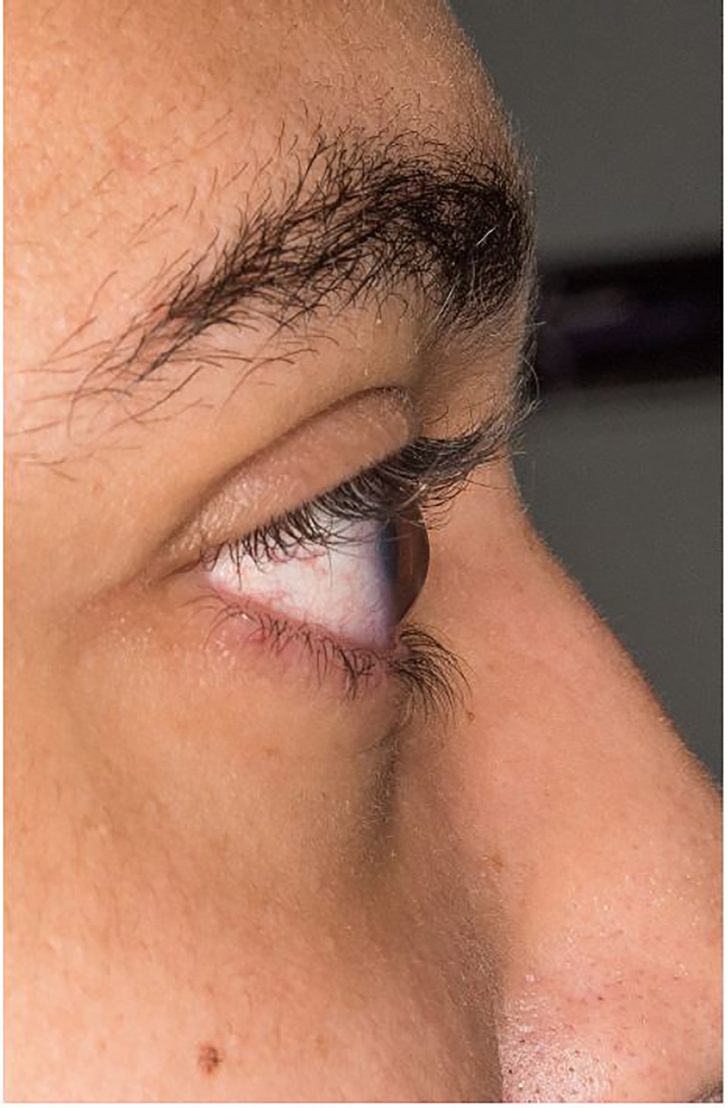
Conical appearance of cornea, seen in keratoconus.

In the lateral view (Image 1) there is a conical appearance of the normally round cornea. This is characteristic of keratoconus, a condition resulting in a thinning and resultant protrusion of the cornea. Distortion of the cornea results in impaired retinal focusing causing myopia and astigmatism. The etiology of the disease is uncertain. Treatment is based on the severity of the condition and ranges from use of rigid contact lenses and collagen cross-linking agents to corneal transplant.^4^

**Image 2:**
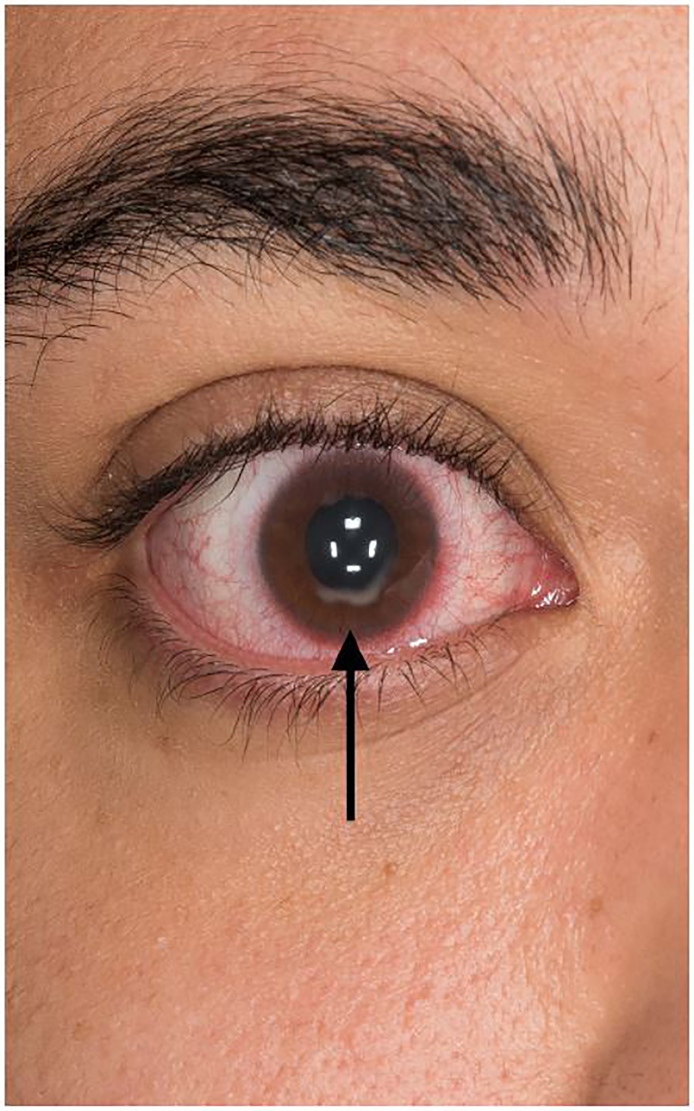
Opacification along the inferior, inner aspect of the iris (arrow), characteristic of corneal hydrops.

The opacification seen along the inferior aspect of the iris (Image 2) is characteristic of corneal hydrops – a collection of aqueous fluid within the cornea through a tear in Descemet’s membrane, which leads to gaping of the posterior surface of the cornea. This allows the aqueous fluid to intrude into the cornea, producing an acute edematous response. Unlike corneal ulcerations, which also have an opacified appearance, in hydrops there is no defect in the outer surface of the cornea and therefore will not uptake fluorescein stain. Patients typically present with a rapid decrease in visual acuity, photophobia and pain. Corneal hydrops is a potential complication seen in patients with corneal ectatic disorders such as keratoconus and post-LASIK ectasia.^1^,^2^ Management includes topical hyperosmotic agents to decrease edema; nonsteroidal anti-inflammatory drugs; corticosteroids and cycloplegics for pain and photophobia; and topical antihistamines if there is an allergic component. 3

CPC-EM Capsule

What do we already know about this clinical entity?

Change in vision, eye discomfort and red eyes are common complaints to the emergency department with multiple different etiologies. The images presented represent a rare cause of such complaints.

What is the major impact of the image(s)?

Keratoconus and corneal hydrops are both diagnoses that can be made in the ED. Diagnosis is made based on visual inspection, requiring no special tests or consultants. Recognizing either the conical cornea or opacification can lead to rapid treatment and outpatient follow-up.

How might this improve emergency medicine practice?

These images will broaden the differential diagnosis for patients with eye complaints. While follow up with ophthalmology provides definitive management, emergency physicians can employ multiple treatment options for symptomatic improvement.
